# Datasets on the corrosion behaviour of nanostructured AISI 316 stainless steel treated by SMAT

**DOI:** 10.1016/j.dib.2019.104033

**Published:** 2019-05-23

**Authors:** Temitope Olugbade

**Affiliations:** Department of Mechanical Engineering, City University of Hong Kong, 83 Tat Chee Avenue, Kowloon Tong, Hong Kong SAR, China

**Keywords:** Corrosion behaviour, 316 SS, Polarization, Potential, Current density, Phase angle

## Abstract

The present paper contains the experimental datasets on the corrosion behaviour of nanostructured AISI 316 stainless steel (SS) through polarization tests in 0.6 M NaCl aqueous solution at room temperature. The nanostructured layers were first obtained through surface mechanical attrition treatment (SMAT) method and the data information on the corrosion behaviour of the nanostructured layer was thereafter collected. Through potentiodynamic polarization tests and electrochemical impedance spectroscopy (EIS) studies, the experimental datasets were obtained to investigate the combined effects of SMAT and low temperature annealing on the corrosion behaviour of 316 SS. The experimental datasets obtained include the open circuit potential (OCP), corrosion current densities, corrosion potential, Nyquist, impedance and phase angle. The datasets provided in this article will be of great help to most aerospace, automobile, and manufacturing industries in determining the actual and practical corrosion behaviour of AISI 316 SS in corrosive and aggressive environment.

**Table:** Specifications table

Subject area	Corrosion
More specific subject area	Corrosion behaviour of materials
Type of data	Figures
How data was acquired	- The experimental datasets were acquired through electrochemical tests using Thales Z3.04 USB electrochemical workstation (Model: IM6, ZAHNER elektrik, Germany). The corrosion experiments were conducted using the conventional three electrode system with platinum (Pt) as the counter electrode, untreated and treated samples as the working electrode, and Ag/AgCl as the reference electrode. - The potential range used is from −100 mV _(Ag/Agcl)_ to +120 mV _(Ag/Agcl)_, with the scan rate of 1 mV/s. For the electrochemical impedance spectroscopy (EIS) study, the frequencies range from 100 MHz to 100 KHz with an amplitude of 10 mV.
Data format	Raw data, analyzed
Experimental factors	The experimental datasets obtained consists of the main corrosion parameters which include open circuit potential (OCP), corrosion current densities, corrosion potential, Nyquist, impedance, and phase angle.
Experimental features	An aqueous solution of 0.6 M NaCl was prepared, after which OCP test was carried out to determine the stability of the samples in the electrolyte before performing polarization and EIS tests. Potentiodynamic polarization measurements of the untreated and treated samples were carried out to determine the corrosion current density (*i*_*corr*_) and potential (*E*_*corr*_). The EIS data was then performed to compare the impedance and phase angle of the untreated sample with the treated ones through SMAT and annealing.
Data source location	City University of Hong Kong, Hong Kong SAR, China.
Data accessibility	Data is available with this article
Related research article	T. O. Olugbade, J. Lu, Enhanced corrosion properties of nanostructured 316 stainless steel in 0.6 M NaCl solution, Journal of Bio- and Tribo-Corrosion, 5, 2019, 38 [1].

**Table:** 

**Value of the data** • Experimental data can be used by most manufacturing industries to determine the actual and practical corrosion behaviour of AISI 316 SS in corrosive and aggressive environment. • The experimental data can be used to test different corrosion models. • The setup, method, results, analysis, and practical relevance can be easily reproduced by other people in different laboratories. • The data obtained on the nanostructured 316 SS with enhanced corrosion properties will give room for future work and improvement.

## 1 Data

**Fig. 1:**
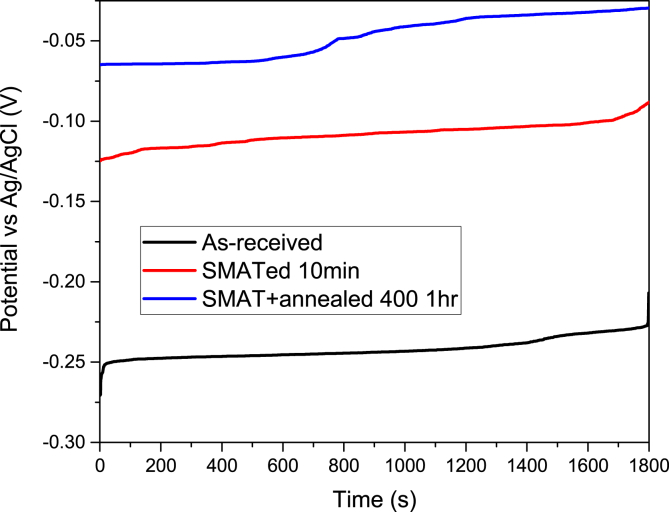
Open circuit potential (OCP) curves of the untreated, SMATed, and SMAT + annealed 316 SS samples immersed in 0.6 M NaCl aqueous solution for 1800s.

**Fig. 2:**
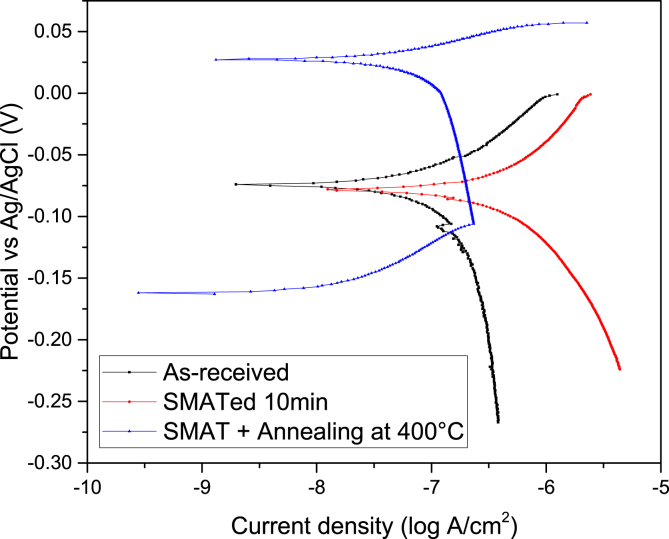
Potentiodynamic polarization curves of the untreated, SMATed, and SMAT + annealed 316 SS samples using Ø2 mm stainless steel balls in 0.6 M NaCl aqueous solution.

**Fig. 3:**
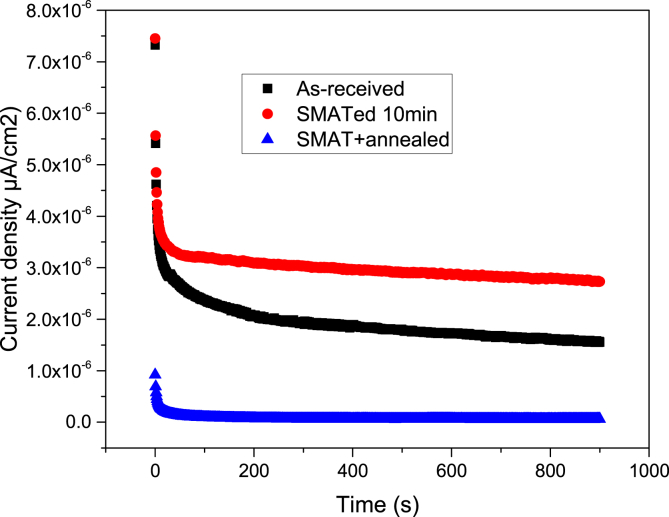
Current-time transients (CTT) of the untreated, SMATed, and SMAT + annealed samples in 0.6 M NaCl aqueous solution at −100mV.

**Fig. 4:**
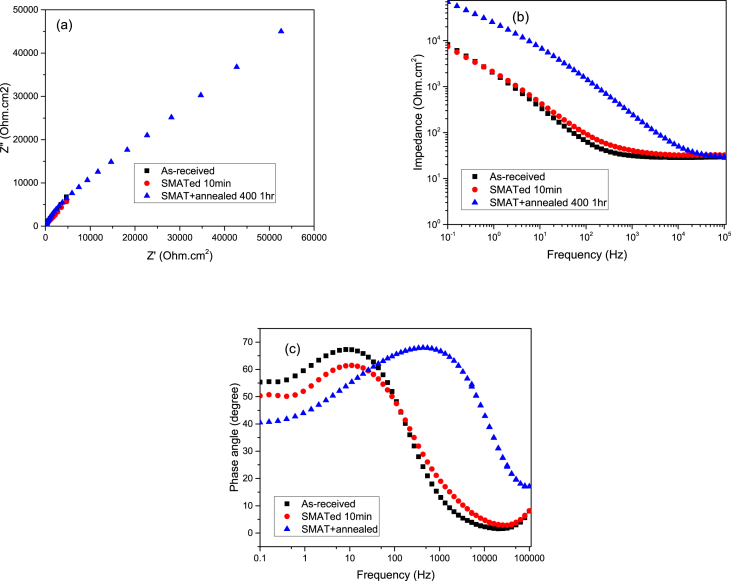
The Nyquist (a) Bode impedance (b) and Bode phase angle (c) plots, of the untreated, SMATed, SMAT + annealed 316 SS samples using Ø2 mm balls in 0.6 M NaCl aqueous solution recorded at their respective OCPs.

**Fig. 5:**
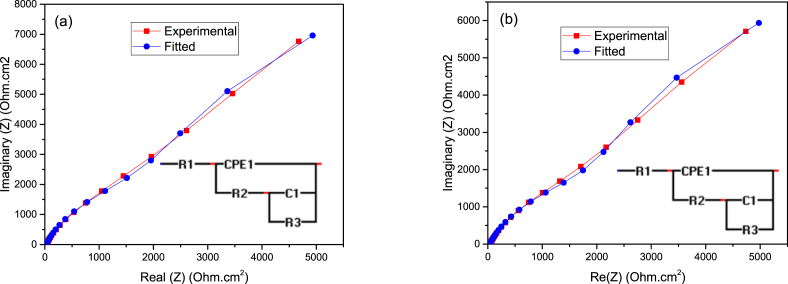
Non-linear least square fitting obtained for the EIS data of the untreated and treated 316 SS samples: (a) untreated; (b) treated for 10 min, (inserts are the equivalent electrical circuit models).

The corrosion properties of nanostructured AISI 316 SS were studied and presented in Fig. 1, Fig. 2, Fig. 3, Fig. 4, Fig. 5. Fig. 1 shows the open circuit potential (OCP) curves of the untreated, treated, treated and annealed 316 SS samples when immersed in 0.6 M NaCl aqueous solution for 1800 s. The polarization curves of the untreated, treated, treated and annealed 316 SS samples for 10 mins using NaCl aqueous solution were presented in Fig. 2. The values of the corrosion potentials (*E*_*corr*_) and current densities (*i*_*corr*_) obtained from the experiment and Tafel extrapolation are compiled. The current–time transients (CTT) at −100 mV _(Ag/AgCl)_ recorded for 900 s for all samples were presented in Fig. 3.

The electrochemical impedance spectroscopy (EIS) curves comprising the Nyquist, impedance, and phase angle plots of the untreated, treated, treated and annealed 316 SS samples using Ø2 mm stainless steel balls for 10 mins in 0.6 M NaCl aqueous solution are shown in Fig. 4a–c, respectively. The treated samples by SMAT and annealing exhibited a much larger diameter of the semi-circle as compared with the untreated one (Fig. 4a). In addition, a markedly increase in impedance was experienced with the treated samples compared to the untreated one (Fig. 4b). Fig. 4c shows the phase angle plots of the untreated and treated 316 SS samples by SMAT and annealing. Fig. 5 show the fittings obtained for the EIS data for both the treated and untreated samples. The inserts are the equivalent electrical circuit models where, R1, R2, and R3 represent the solution resistance, film resistance, and charge transfer resistances, respectively. C1 is the double layer capacitance while CPE1 represents the constant phase element. The better non-linear least square fitting of the experimental data was obtained within 5% error [2] using EIS spectra analyzer software, with chi-square goodness of fit values (r^2^ amplitude) of 0.003 and 0.010 for the untreated and treated samples respectively.

## 2 Experimental design, materials, and methods

AISI 316 SS plate with dimension 70 × 70 × 1 mm^3^ was used in this study. The samples were cut into sizes using electrical discharge machining (EDM). For the corrosion test, an aqueous solution of 0.6 M NaCl was used as the corrosive medium, with Platinum (Pt), Ag/AgCl and samples as the counter, reference and working electrodes, respectively. The samples were treated on both surfaces with spherical 304 SS balls of Ø2 mm at room temperature for 10 min using SMAT technique. The SMAT method, which is preferable to other methods such as coating/deposition [3], [4] in retaining the inherent properties of materials after treatment, has been extensively described in the literature [5], [6] with application in aircraft, oil and gas, etc. [7], [8], [9], [10]. After SMAT, the treated surface was slightly polished to reduce the effect of oxidation and surface roughness on the corrosion behaviour.

The corrosion behaviour of untreated and the treated 316 SS samples was investigated by electrochemical tests in 0.6 M NaCl. For each of the three conditions (untreated, treated, treated and annealed), three different samples were used to measure the corrosion properties and the resulting results were compared. To ensure reproducibility of the experiment results, the electrochemical tests were repeated for three times. To release the high strain energy induced during SMAT, the treated 316 SS sample was subjected to a low temperature annealing treatment at 400 °C for 1 h. The resulting potentiodynamic polarization curve with the corrosion potential (*E*_*cor*r_) and corrosion current density (*i*_*corr*_) is shown in Fig. 2. The corrosion potential (*E*_*cor*r_) significantly increased from −0.063 V to 0.026 V while the corrosion current density (*i*_*corr*_) decreased from 1.08 mA/cm^2^ to 0.53 mA/cm^2^.

To have knowledge about the impedance, an EIS analysis was carried out and the treated samples exhibited a much larger diameter of the semi circles as compared with the untreated one. The (supplementary material) contains the full datasets of the potentiodynamic polarization and electrochemical impedance spectroscopy (EIS) studies.
