# Supplementary material for: Datasets on the corrosion behaviour of nanostructured AISI 316 stainless steel treated by SMAT
Source: Data Brief. 2019 May 23;25:104033. doi: 10.1016/j.dib.2019.104033 (PMC6554219; doi:10.1016/j.dib.2019.104033)
Supplement: Supplementary file 1 — Multimedia component 1 [file mmc1.docx]

**CONFLICT OF INTEREST**

Manuscript title - Datasets on the corrosion Behaviour of Nanostructured AISI 316 Stainless Steel Treated by SMAT

I wish to confirm that there are no known conflicts of interest associated with this article. I also confirm that there are no other persons who satisfied the criteria for authorship but are not listed.

Thank you.

Signed

Temitope Olugbade

April 26, 2019.
